# Prevalence, Clinical Aspects and Outcomes in a Large Cohort of Persons with Diabetic Foot Disease: Comparison between Neuropathic and Ischemic Ulcers

**DOI:** 10.3390/jcm9061780

**Published:** 2020-06-08

**Authors:** Marco Meloni, Valentina Izzo, Laura Giurato, José Luis Lázaro-Martínez, Luigi Uccioli

**Affiliations:** 1 Diabetic Foot Unit, Department of Medicina dei Sistemi, University of Tor Vergata, 00133 Rome, Italy; valentina_izzo@virgilio.it (V.I.); lauragiurato@yahoo.it (L.G.); luccioli@yahoo.com (L.U.); 2 Diabetic Foot Unit, Facultad de Medicina, Universidad Complutense de Madrid, Instituto de Investigacion Sanitaria de Hospital Clinico San Carlos (IdISSC), 28040 Madrid, Spain; diabetes@ucm.es

**Keywords:** diabetes, diabetic foot ulcers, co-morbidities, wound healing, amputation

## Abstract

This study aims to evaluate clinical and ulcer characteristics as well the outcomes of patients with diabetic foot ulcers (DFUs). The study group was composed of DFUs patients managed by a limb salvage protocol according to guidance. Clinical and ulcers findings were described, and 1-year outcomes defined as limb salvage, healing, healing time, major amputation and death were compared between neuropathic and ischemic DFUs. One thousand, one hundred and ninety-eight subjects were included; 386 (32.2%) neuropathic and 812 (67.8%) ischemic DFUs. Neuropathic patients were younger (69.5 ± 11.5 vs. 74.5 ± 11.5, *p* < 0.0001) and reported less cases of nephropathy (22.8 vs. 39.6%, *p* < 0.0001), ischemic heart disease (22.8 vs. 36.9, *p* = 0.0004), cerebrovascular disease (8.3 vs. 17.2%, *p* = 0.002), heart failure (10.1 vs. 24.7%, *p* = 0.0002) and end-stage-renal-disease (ESRD) (5.4 vs. 27%, *p* = 0.0001) than ischemic patients; they also showed less cases of large (>5 cm^2^) (10.3 vs. 22.9%, *p* = 0.0007), infected (40.4 vs. 55.7%, *p* = 0.0005) and deep to the bone (22.3 vs. 39.2, *p* = 0.0002) ulcers, as well less multiple ulcerations (21.8 vs. 32.8%, *p* = 0.006) than patients with ischemic DFUs. The outcomes for neuropathic and ischemic DFUs were limb salvage (98.4 vs. 82.3%, *p* < 0.0001), healing (97.3 vs. 79.6%, *p* < 0.0001), healing time (34.9 vs. 35.6 weeks, *p* = 0.8), major amputation (0.5 vs. 6.6%, *p* = 0.0001), death (1.1 vs. 11%, *p* < 0.0001) respectively. Revascularization failure and ESRD were independent predictors of major amputation, while heart failure and number of co-morbidities (≥5) were independent predictors of death. Ischemic DFUs patients showed more severe clinical and ulcers features as well worse outcomes than neuropathic DFUs patients.

## 1. Introduction

Diabetic foot disease (DFD) is the most severe consequence of two diabetes related long-term complications: peripheral neuropathy (PN) and peripheral arterial disease (PAD). Foot ulceration is usually the main clinical expression of DFD [1]. Diabetic foot ulcers (DFUs) affect up to 15% of the diabetic population at some time in their life and they represent the first cause of hospitalization, minor and major amputation among diabetic subjects [2,3].

Patients with DFD are often very fragile and foot ulceration may be just a part of an extremely complex clinical condition in which specific long-term complications (PN and PAD) and concomitant co-diseases affect the general health of patients. A 5-year mortality rate was reported following new-onset of DFU, which is between 25% and 60% [4,5] higher than several types of cancers. Moreover, cardiovascular and renal disease are the main causes of death. Diabetic foot (DF) patients have reported a complex interplay of several inflammatory markers which can affect cardio-vascular system and DF influence a faster progression of cardio-vascular damage and morbidity [6,7]. Therefore, not only does DFD require early management of foot ulceration but also the assessment of all comorbidities that may influence the outcomes.

Within this framework, it is necessary to consider two patterns of DFUs in patients with or without peripheral arterial disease (PAD), termed respectively as neuro-ischemic/ischemic ulcers and neuropathic ulcers. It has been reported that until now, in developed countries, the rate of PAD in patients with foot ulceration is approximately 50% [8,9], while neuropathic ulcers are more prevalent in low income countries [10,11].

PAD increases the risk of non-healing and major amputation [12,13,14], and it is associated with an increased risk of concomitant cardiovascular disease, as well as ischemic heart disease, cerebrovascular disease, and chronic kidney disease, and a high risk of mortality [15,16]. Therefore, it is essential that PAD in patients with DFUs is recognized early and managed accordingly.

Based on their daily experience, the authors retain that a deep understanding of the patients DFU history, management and outcomes could lead to an improvement in our strategies.

This study aims to evaluate the pattern of diabetes-related complications and co-morbidities in patients with DFUs, comparing neuropathic and ischemic patients. Furthermore, the characteristics of neuropathic and ischemic/neuro-ischemic DFUs will be reported and compared, as well as the long-term outcomes.

## 2. Materials and Methods

Consecutive patients who were referred to our diabetic foot unit for a new diabetic foot problem between January 2010 and December 2018 were considered for this study. Patients included were those attending the clinic for a new foot ulceration, including both neuropathic and ischemic/neuro-ischemic DFUs. Subjects referred with an unsalvageable foot condition requiring major amputation, those with a life expectancy of less than 6 months and those who lost to follow-up during the first 12 months were excluded.

All patients included were managed through a pre-set limb salvage protocol including revascularization in the case of ischemic/neuro-ischemic ulcers, antibiotic therapy and surgical treatment for infected wounds, dedicated off-loading, appropriate wound care, and management of diabetes and comorbidities according to guidance [17,18].

Data were collected in a local database and retrospectively analyzed. Baseline demographic, clinical and ulcer findings were recorded.

The study has been done and approved according to local ethics committee policy. At admission, patients provided their verbal consent.

### 2.1. Microvascular Complications

Diabetic retinopathy, neuropathy, and nephropathy were conditions reported. Retinopathy was considered in the case of proliferative or not proliferative retinopathy; peripheral neuropathy was considered in the case of loss of peripheral sensitivity detected through vibration perception (128 Hz tuning fork) or Semmes-Weinstein 10-g monofilament [17,18]; nephropathy was considered in the case of albuminuria, both micro (30–300 mcg/mg creatinine) and macroalbuminuria (>300 mcg/mg creatinine) [19].

### 2.2. Macrovascular Complications

Ischemic heart disease (IHD) was considered in the case of previous acute coronary syndrome or coronary revascularization, evidence of angina, significant changes on electrocardiography (above or under-leveling ST, q wave, inversion of T wave, new left bundle branch block). Cerebrovascular disease was considered in the case of previous cerebrovascular ischemia, previous carotid revascularization, or significant carotid artery disease (occlusion >70%).

### 2.3. Comorbidities

Hypertension was considered in the case of blood pressure >130/80 mmHg persistently or current antihypertensive therapy [19]; hypercholesterolemia was defined as low density lipoproteins (LDL) > 70 mg/dL or needing statin therapy [19]; heart failure (HF) was considered in the case of typical symptoms and signs of HF reduced left ventricular ejection fraction (LVEF) (<40%) or normal or only mildly reduced LVEF and elevated levels of brain natriuretic peptides (BNP > 35 pg/mL and/or NT-proBNP > 125 pg/mL) without dilated left ventricle (LV), associated with relevant structural heart disease (LV hypertrophy/left atrial enlargement) and/or diastolic dysfunction [20]. End-stage-renal-disease (ESRD) requiring dialysis was considered in the case of chronic renal replacement therapy. Anemia was considered according to hemoglobin values at the first assessment. Patients were only considered smokers if they had a smoking habit at the time of treatment.

### 2.4. Ulcer Features

Baseline ulcer characteristics (location, size, depth, infection) reported at the first assessment were recorded. Infection was considered in the case of clinical signs according to International Working Group on the Diabetic Foot (IWGDF) [17,18]. Ulcer was considered deep to the bone in the case of bone exposure or positive probe-to-bone test. The site of ulcer location was characterized as forefoot, midfoot and rearfoot; in the presence of more than one ulcer, it was considered multiple location.

Neuropathic ulcers were considered in the case of patients with PN without PAD; ischemic ulcers were considered in the case of patients with PAD, regardless of the presence or not of PN. PAD was considered in the case of the absent pulses and ankle-brachial index of <0.9 or TcPO2 < 50 mmHg, in addition to evident stenosis and/or an obstruction at duplex ultra-sound [18,19,20].

### 2.5. Outcomes

Micro and macrovascular diabetes-related complications, co-morbidities, ulcers characteristics and outcomes in patients with neuropathic and ischemic DFUs were reported and compared.

Limb salvage, healing, healing time, amputation, and mortality after 1-year of follow-up were the primary outcomes considered. Limb salvage was considered in the case of healing or incomplete healing in patients with preserved limb function; healing was considered in the case of complete epithelialization of previous ulceration during the follow-up; healing time was considered as the time reported in weeks which occurred from the first assessment and the complete epithelialization; amputation was considered as any amputation above-the-ankle and included below and above-the-knee.

As secondary endpoint, the association between the number of comorbidities and limb salvage in the whole population of neuropathic and ischemic subjects was reported.

All potential predictors of major amputation and death where evaluated.

### 2.6. Statistical Analysis

Statistical analysis was performed by SAS (JMP12; SAS Institute, Cary, NC, USA) for personal computer. Data are expressed as means ± SD. Comparison between groups were reported by the Student’s *t* test (frequency data) or ANOVA (continuous data). Univariable logistic regression analysis was performed with all potential predictor variable with the outcome of interest (major amputation and death). Then, all positive predictors were entered simultaneously in a multivariate logistic regression model. These models yielded a set of variables that best predict the outcome of interest. *p* < 0.5 was considered statistically significant.

## 3. Results

One thousand, one hundred and ninety-eight patients were included. The mean age was 73 ± 12 years; 758/1198 (63.3%) were males, 1130/1198 (94.3%) had type 2 diabetes and the mean HbA1c was 62 ± 24 mmol/mol (Table 1).

Three hundred eighty-seven (387) (33.2%) patients had neuropathic DFUs while 812 (67.8%) patients had ischemic/neuro-ischemic DFUs. Ischemic DF patients were older, had a longer duration of diabetes and higher baseline HbA1c values when compared to neuropathic DF subjects (Table 1).

They were characterized by the high presence of long-term diabetes-related complications, mainly peripheral neuropathy (92%), retinopathy (51%), nephropathy (34%), PAD (68%) and ischemic heart disease (32%). Regarding concomitant co-morbidities, they frequently reported hypertension (79%), dyslipidemia (40%), and often ESRD requiring dialysis (22%) and heart failure (20%) (Table 2).

### 3.1. Microvascular and Microvascular Complications, and Comorbidities

Ischemic DF patients had more diabetic nephropathy and less peripheral reduced sensitivity than neuropathic DF subjects (Table 2).

Ischemic DF patients showed a higher rate of ischemic heart disease and cerebrovascular disease than neuropathic subjects (Table 2).

Ischemic DF patients showed more cases of HF, ESRD and anemia than neuropathic persons. No difference was recorded in terms of hypertension, dyslipidemia and smoking between the two groups (Table 2).

Overall, 16.5% of patients reported 4 co-morbidities concurring with diabetes and 10.8% had 5 or more. More patients with ischemic DF presented with 4 or more concomitant co-morbidities than patients with neuropathic DF (Table 3).

### 3.2. Ulcers Features

Ischemic DF patients showed more cases of multiple lesions in comparison to neuropathic DF patients, while neuropathic DF subjects showed more cases of forefoot localization than ischemic DF patients. Ischemic DFUs were larger, more infected and deep to the bone in more cases than neuropathic DFUs (Table 4).

### 3.3. Outcomes

**Figure 1:**
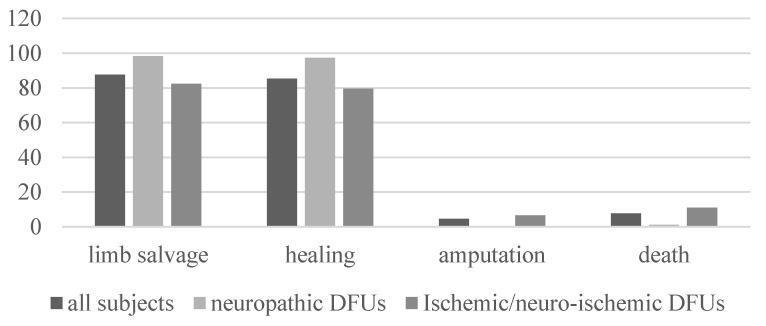
Outcome of all subjects, neuropathic and ischemic/neuro-ischemic patients.

One thousand and fifty (1050) (87.7%) patients had limb salvage after 1-year of follow-up, 1022 (85.3%) patients healed in an average time of 35.4 weeks (range 31.9–39.1), 55 (4.6%) were amputated (major amputation), and 93 (7.7%) died (Figure 1).

The outcomes for neuropathic and ischemic patients were, respectively: limb salvage (98.4 vs. 82.4%, *p* < 0.0001), healing (97.3 vs. 79.6%, *p* < 0.0001), average healing time (34.9 vs. 35.6 weeks, *p* = 0.8), amputation (0.5 vs. 6.6%, *p* = 0.0001), and death (1.1%vs. 11%, *p* < 0.0001) (Figure 1).

In the whole population, the rate of limb salvage gradually decreased as the number of concomitant co-morbidities increased; furthermore, the rate of limb salvage in persons with ischemia was less than those with neuropathy, despite having a similar number of coexisting diseases (Table 5).

At the multivariate analysis of all predictors found at univariate analysis, revascularization failure and ESRD were independent predictors of major amputation, while heart failure and the presence of five or more concomitant co-diseases were independent predictors of mortality (Table 6).

## 4. Discussion

This study offers a complete overview on ulceration findings, clinical characteristics, and long-term outcomes in a large cohort of diabetic foot patients including those with both neuropathic and neuro-ischemic/ischemic DFUs.

Overall, 87.7% had limb salvage and 85.3% of patients healed in an average time of 35.4 weeks, while 4.6% had major amputation and 7.7% died.

On the one hand, the reported data are very encouraging because through a specialized multi-disciplinary team approach, we achieved a great rate of limb salvage and healing, mainly in neuropathic subjects. On the other hand, we observed a significant increase in ischemic DFUs, which were approximately two times higher than neuropathic DFUs and, among those reported, showed a higher rate of amputation (6.6 vs. 0.5%) and death (11 vs. 1.1%), and a lower rate of healing (79.6 vs. 97.3%) in comparison to neuropathic subjects.

These data are partially similar to those reported by the Eurodiale study [12] which showed that ischemic patients had a higher rate of non-healing in comparison to neuropathic patients; the same Eurodiale study described ischemic DFUs as larger, deeper and more infected than neuropathic DFUs, as we found in this current cohort. In addition, we reported that in most cases of ischemic DF, there was evidence of heel ulceration and multiple ulcers, while in neuropathic patients, there was higher involvement of the forefoot than in ischemic patients.

The ulcer size and the presence of infection could increase the risk of non-healing [21]. Additionally, infection is a predictor of amputation [22] and mortality in frail patients with PAD [23]; furthermore, heel ulcers could influence both non-healing and amputation [24,25].

Patients included in our study group were elderly (mean age >70 years), with a long diabetes duration (approximately 24 years) and poor glycemic control. They were characterized by the high presence of microvascular and macrovascular complication, and several concomitant co-morbidities, such as hypertension, dyslipidemia, ESRD and heart failure.

Patients with ischemic ulcers were older than those with neuropathic ulcers and showed higher rates of microvascular (40% with nephropathy) and macrovascular (37% with ischemic heart disease and 17% with carotid artery disease) complications, and concomitant co-morbidities (82% with hypertension, 27% on dialysis, 25% with heart failure and 24% with anemia) in comparison to patients with neuropathic ulcers.

In the whole population, approximately 16% of patients reported at least four concomitant co-morbidities, and 10.8% had five or more. Patients with ischemic DF were more likely to present with four or more concomitant co-morbidities than patients with neuropathic DF (32 vs. 16%). It is also very interesting to highlight the rate of limb salvage decreases with the increase in the number of co-morbidities. However, the limb salvage rate was 100% and 90%, respectively, in subjects without and with three concomitant co-diseases or fewer. Nonetheless, the rate of limb salvage is significantly lower in those with ischemic DF than neuropathic DF despite the similar number of co-morbidities. These data are already evident in the presence of one co-disease (100% of limb salvage in neuropathic patients and 88% in ischemic patients) and much more evident in the presence of three or more co-diseases, suggesting that co-morbidities in patients with PAD reduce the possibility of limb salvage, which is probably due to the impact of PAD perse.

The role of co-morbidities is reinforced by the multivariate analysis which revealed that ESRD was an independent predictor of major amputation and heart failure, and, additionally, the number of concomitant co-diseases (≥5) were independent predictor of mortality.

The role of co-morbidities in DFD is often underestimated, even though it is well known that they can significantly influence outcomes for patients with DFUs. It is maintained that ulcer-related outcomes may underestimate the morbidity and mortality associated with DFD [26], and recently clinicians are more often focused on the patient’s comorbidities in the management of DFD.

Our data highlight that DF should be considered as a marker of multi-organ disease in ischemic subjects, with a significant impact on outcomes. Many papers have reported that co-morbidities, mainly dialysis and heart failure, increase the risk of major amputation and outcomes [23,27].

Patients with DFUs, mainly ischemic, are extremely difficult to treat, and foot injury is often just a part of a very complex clinical condition. Ischemic patients often require hospitalization to be treated for revascularization and due to the presence of severe co-morbidities, in some cases, a fast limb salvage protocol may not only address limb salvage, but may also save the patient’s life.

Similarly, the Eurodiale study showed that heart failure and ERSD had a greater incidence in patients with PAD, and in addition, ESRD was an independent predictor of non-healing [12].

Gershater et al., in a prospective study on 1148 hundred and eighty patients, reported that the absence of uremia and heart disease were clinical factors related to primary healing in the whole population and in survivors. Conversely, diabetic nephropathy and uremia were predictors of non-healing in survivor patients with ischemic/neuro-ischemic ulcers, and uremia was related to major amputation in ischemic ulcers. Furthermore, deceased patients showed more ischemic ulcers and more co-morbidities than the other groups [28].

Alpeqvist et al. showed that Creatinine values <130 μmol/L and the absence of congestive heart failure were independent predictors of primary healing in a population composed of 1115 patients with ischemic DFUs [29].

Faglia et al. showed that ischemic heart disease was the leading cause of death; dialysis and a history of cardiac disease were independent predictors of death; dialysis was an independent predictor of major amputation in 554 patients with DFUs and CLI when treated using a limb salvage protocol, which includes revascularization [23].

In our previous experience, we observed that dialyzed patients had higher risk of non-healing and major amputation than subjects with preserved renal function [30], and that patients with ischemic DFUs affected by heart failure and dialysis had a very high risk of one-year mortality (56%) [23].

Therefore, co-morbidities, such as heart disease, including both coronary artery disease and heart failure, and ESRD not only reduce the chance of healing, but they are also independent predictors of mortality.

In the current study, we confirm that ischemic heart disease is quite typical in diabetic foot patients with PAD. It has already been reported that approximately 50% of diabetic patients with PAD have a concomitant ischemic heart disease [31]. Heart impairment is common in patients with DFUs, even in asymptomatic persons. Londhal et al. reported that patients with chronic DFUs, in 69% of cases, presented with myocardial infarction and/or hypertension and/or heart failure; in 78% left ventricular dysfunction and/or hypertrophy and/or diastolic dysfunction, and in 76% echocardiographic signs of heart dysfunction without any previous history of cardiovascular disease [32].

Therefore, patients with DFUs, mainly ischemic DFUs, should be considered as subjects with a high risk of heart disease—mainly heart failure secondary to ischemic heart disease.

It is noteworthy that micro/macrovascular diabetes-related complications were more frequent in patients with ischemic DF than neuropathic DF, although a similar diabetes duration was reported. Therefore, it may be evident that age, concomitant co-morbidities (e.g., hypertension and renal impairment), poor glycemic control and individual susceptibility could increase the risk of developing PAD and the abovementioned complications.

It is also necessary to highlight that revascularization failure was an independent predictor of major amputation. Revascularization failure was considered as technical recanalization failure of occluded vessels (defined as the impossibility to overcome the obstruction) and/or absence of arterial flow to the foot. On the one hand this data suggests that the severity of PAD could negatively influence the revascularization procedure and outcome, but on the other hand also confirms that failed revascularization is a predictor of major amputation as already reported by Faglia et al. [27] and our research group in a recent study [33].

Therefore, DFD is a complex clinical condition characterized by foot injury, severe patterns of PAD, and several concomitant co-morbidities which could influence management and outcomes. The presence of a foot ulceration in diabetics should always be considered a strong risk factor for early and long-term mortality, mainly in ischemic subjects which have shown 5-year-mortality, of approximately 60% [34]. It is evident that persons with DFUs are often fragile and the onset of foot ulceration could worsen their frail health status.

This study gives a complete overview on clinical and ulcers features, and the long-term outcomes in a very large cohort of patients with DFUs, which proves to identify specific characteristics in the current pattern of neuropathic and ischemic subjects. To the best of our knowledge it is the first study after the Eurodiale study to evaluate and compare the prevalence, characteristics and outcomes of neuropathic and ischemic DF patients in a very large cohort of patients; furthermore, it is the first to highlight that the number of comorbidities could influence the possibility of limb salvage, in addition to the fact that concomitant co-diseases may have a major influence on ischemic rather than neuropathic DFUs.

The current study is a retrospective study and data were collected from one single diabetic foot center and, accordingly, outcomes are related to our comprehensive limb salvage protocol, performed by an expert multidisciplinary diabetic foot team. Future research may be useful to identify if outcomes are influenced only by the number of comorbidities or by a comorbidity score, whereby each concomitant disease has a specific burden.

## 5. Conclusions

Our data illustrate that among current patients affected by DFD, there is a prevalence of ischemic DFUs in comparison to neuropathic DFUs, and there also seems to have been an increase in ischemic subjects over the last years. Patients with DFUs are complex subjects, who in addition to foot injury, are often older in age and display several diabetes-related complications and comorbidities—mainly cardiovascular. This study reinforces the concept that patients with ischemic DFUs report more severe wound and clinical features than those with neuropathic DFUs, and that there is a lower rate of limb salvage in spite of the similar number of concomitant co-diseases. Furthermore, co-morbidities appear to play a key role in the outcomes of patients with DFD.

While aiming to reduce amputation and mortality, DFD should be considered a multi-organ disease, which means that patients need an intensive global and multidisciplinary treatment plan with close control of cardiovascular risk factors.

## Figures and Tables

**Table 1:** Demographic and diabetes-related characteristic of all subjects, neuropathic and ischemic foot patients.

Variables	*n* = 1198	Neuropathic DF (*n* = 386)	Ischemic DF (*n* = 812)	*p*
Age (years)	72.9 ± 11.8	69.5 ± 11.5	74.5 ± 11.5	<0.0001
Sex (man)	758 (63.3%)	(248) 64.2%	(510) 62.8%	0.7
Diabetes (type 2)	1130/1198 (94.3%)	(360) 93.2%	(770) 94.8%	0.4
Diabetes duration (years)	24 ± 12	24 ± 11	24 ± 12	0.7
HbA1c (mmol/mol)	62 ± 24	59 ± 22	62 ± 25	0.1

**Table 2:** Microvascular and Macrovascular related complications, and concomitant comorbidities.

Microvas. Complications	*n* = 1198	Neuropathic DF (*n* = 386)	Ischemic DF (*n* = 812)	*p*-Value
Retinopathy	612 (51.2%)	194 (50.2%)	420 (51.7%)	0.7
Nephropathy	410 (34.2%)	88 (22.8%)	321 (39.6%)	<0.0001
Peripheral neuropathy	1102 (92%)	386 (100%)	716 (88.2)	0.0002
Macrovas. Complications				
Ischemic heart disease	388 (32.4%)	88 (22.8%)	(299) 36.9%	0.0004
Cerebrovascular disease	192 (14.3%)	8.3%	(140) 17.2%	0.002
Peripheral arterial disease	812 (67.8%)	–	812 (100%)	
Comorbidities				
Hypertension	950 (79.3%)	284 (73.6%)	666 (82%)	0.01
Dyslipidemia	482 (40.2%)	168 (43.5%)	314 (38.7%)	0.2
Heart failure	269 (22.4%)	68 (10.1%)	201 (24.7%)	0.0002
ESRD	240 (20%)	21 (5.4%)	219 (27%)	0.0001
Anemia	226 (18.8%)	31 (8%)	195 (24%)	0.0003
Smoke	90 (7.5%)	24 (6.2%)	66 (8.1%)	0.4

Microvasc.: microvascular; macrovasc.: macrovascular; ESRD: end-stage-renal-disease.

**Table 3:** Rate of co-diseases in all subjects, neuropathic and ischemic DF groups.

Co-diseases (*n*) (%)	*n* = 1198	Neuropathic DF (*n* = 387)	Ischemic DF (*n* = 812)	*p*-Values
0 co-disease	70 (5.8%)	34 (8.8%)	36 (4.4)	0.1
1 co-disease	190 (15.8%)	72 (18.6%)	118 (14.5%)	0.2
2 co-disease	206 (25.5%)	94 (24.3%)	212 (26.1)	0.8
3 co-disease	298 (24.9%)	122 (31.6%)	176 (21.7%)	0.06
4 co-disease	198 (16.5%)	46 (11.9%)	152 (18.7%)	0.005
≥5 co-disease	136 (10.8%)	18 (4.6%)	118 (13.6%)	0.0003

**Table 4:** Ulcer baseline characteristics in all subjects, neuropathic and ischemic DF patients.

Characteristics	*n* = 1198	Neuropathic DF (*n* = 386)	Ischemic DF (*n* = 812)	*p*-Value
Size (>5 cm^2^)	112 (18.7%)	40 (10.3%)	186 (22.9%)	0.0007
Depth (to the bone)	404 (33.7%)	86 (22.3%)	286 (39.2%)	0.0002
Infection	608 (50.7%)	156 (40.4%)	452 (55.7%)	0.0005
Ulcer location				
Forefoot	700 (58.4%)	262 (67.9%)	438 (53.9%)	0.002
Midfoot	62 (5.2%)	16 (5.2%)	42 (5.2%)	0.1
Rearfoot	86/1198 (7.2%)	16 (5.2%)	66 (8.1%)	0.6
Multiple ulcers (>1)	350/1198 (29.2%)	84 (21.8%)	266 (32.8%)	0.006

**Table 5:** Rate of limb salvage according to the number of co-disease in all subjects, neuropathic and ischemic DF groups.

Co-Diseases (%)	All Subjects	Neuropathic DF	Ischemic DF	*p*-Values
0 co-disease	100%	100%	100%	
1 co-disease	92.4%	100%	87.9%	0.008
2 co-disease	90.1%	100%	85.7%	0.0003
3 co-disease	89.7%	100%	82.8%	<0.0001
4 co-disease	78.8%	95.6%	77%	0.01
≥5 co-disease	77.9%	88.8%	73.7%	0.001

**Table 6:** Multivariate analysis of independent predictors of outcome (major amputation and death) found at univariate analysis.

Variables	Major Amputation			Death		
	OR	95% CI	*p*-Value	OR	95% CI	*p*-Value
Ulcer size (>5 cm^2^)	1.1	0.7–1.6	0.2			
Heel ulcer	1.2	0.8–1.8	0.08			
Infection	0.9	0.6–1.4	0.6	1.4	0.9–1.7	0.06
Revascularization failure	5.7	2.9–11.5	0.001			
ESRD	2.1	1.7–5.8	0.02	0.8	0.5–1.9	0.1
IHD				1.5	0.8–2.2	0.07
Heart failure				6.4	2.1–14.5	0.0001
Number of co-diseases (≥5)				3.4	1.8–7.7	0.0001

ESRD: end-stage-renal-disease; IHD: ischemic heart disease.
